# Listening Effort, Sleep Quality, and Digital Addiction: Exploring Their Links

**DOI:** 10.1002/brb3.71539

**Published:** 2026-06-04

**Authors:** Banu Baş, Elif Zehra Biber, Gülse Akdemir

**Affiliations:** ^1^ Faculty of Health Sciences, Department of Audiology Ankara Yıldırım Beyazıt University Ankara Türkiye; ^2^ Department of Audiology Institute of Health Sciences Ankara Yıldırım Beyazıt University Ankara Türkiye

**Keywords:** effortful listening, mental fatigue, sleep deprivation, technology overuse

## Abstract

**Objective:**

This study aimed to clarify the relationship between sleep quality and listening effort, as well as the relationship between digital addiction and listening effort.

**Methods:**

Listening effort was assessed via a dual‐task paradigm. In this paradigm, repeating the auditorily presented sentence was designated the primary task, whereas a modified version of the Stop Signal Task served as the secondary task. Sleep quality was evaluated via the Pittsburgh Sleep Quality Index (PSQI), and digital addiction was assessed via the Digital Addiction Scale (DAS).

**Results:**

No significant differences were detected between the PSQI groups in terms of the reaction time dual‐task effect (RT‐DTE) or the error rate dual‐task effect (ER‐DTE), and no significant relationships were detected between the PSQI score and either the RT‐DTE or the ER‐DTE. Similarly, no correlation was found between the DAS and RT‐DTE or ER‐DTE.

**Conclusion:**

This study is the first to explore listening effort in relation to sleep quality and digital addiction. The results showed no significant effects of either variable on listening effort in healthy young adults under controlled conditions. These findings suggest that listening effort may be resilient to variations in sleep quality and digital addiction, and that other cognitive or auditory factors may play a more critical role. Further studies with more comprehensive designs are needed to clarify these relationships.

## Graphical abstract

This study examined the relationships between sleep quality, digital addiction, and listening effort in young adults. Findings revealed no significant associations, suggesting that listening effort may remain stable despite variations in sleep and digital habits, emphasizing the role of other cognitive and auditory factors in listening performance.

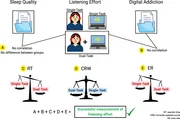

## 1 Introduction

Listening effort refers to the cognitive resources required to attend to and comprehend auditory input under challenging listening conditions (Francis and Love 2020). In addition to speech perception, skills such as sound localization and auditory discrimination can also contribute to listening effort (Escobar et al. 2019). As listening conditions become more demanding, the use of cognitive resources increases. Listening effort is influenced by cognitive resources such as attention, working memory, and executive functions (Francis and Love 2020). Motivation is another factor that influences listening effort. Even when auditory demands increase, low motivation may reduce or limit the extent to which individuals engage in listening effort (Pichora‐Fuller et al. 2016).

Sleep has an important influence on cognitive and physical health, as well as general physiological functioning (Mah et al. 2018). Sleep quality is a concept that reflects whether sleep is restorative and supports an individual's physical and mental health. Good sleep quality is not solely determined by sleep duration. Difficulties experienced during sleep, subjective perceptions, and the extent to which these factors affect daily functioning all contribute to the overall assessment of sleep quality. Poor sleep quality can negatively impact cognitive functions, learning and memory, reaction time, auditory alertness, and mood (Van Dongen et al. 2003; Walker and Stickgold 2004). Since listening effort relies heavily on attention and working memory, it is plausible that reduced sleep quality may increase the amount of effort required to maintain performance in auditory tasks.

Sleep quality can be influenced by factors such as age, physical activity, nutrition style, and lifestyle. Studies suggest that between 30% and 70% of university students experience poor sleep quality (Schmickler et al. 2023). In addition to the primary factors affecting sleep quality in university students, digital addiction and problematic internet use are also significant contributors (Wang and Bíró 2021; Zhu et al. 2024). The widespread use of digital platforms, particularly during late‐night hours, may lead to delayed sleep onset, reduced sleep duration, and impaired daytime alertness (Chang et al. 2015). Moreover, excessive digital use is linked to mental fatigue, reduced attention span, and poorer executive functioning (Demirci et al. 2015). These cognitive outcomes suggest a potential indirect pathway through which digital addiction may also influence listening effort by taxing the cognitive resources needed for effective listening. Attention and working memory, which are cognitive resources affecting listening effort, are associated with the frontal lobe (Naghavi and Nyberg 2005). Sleep deprivation can lead to poor sleep quality and negatively impact frontal lobe function, potentially resulting in impaired attention and working memory (Alhola and Polo‐Kantola 2007).

Driven by the ongoing progress of technology, engagement with digital platforms has emerged as an integral part of everyday life. Frequent use of digital devices can lead to distractions, difficulties in maintaining focus, and mental fatigue. These issues may pose risks that negatively impact both physical and mental health. Among university students, excessive exposure to smartphones has been associated with increased levels of anxiety and depression, which in turn can result in poor sleep quality (Demirci et al. 2015). The blue light emitted from screens suppresses the production of melatonin, a hormone that regulates sleep, thereby delaying the onset of sleep (Chang et al. 2015). Exposure to digital devices late at night may decrease sleep quality and impair cognitive performance.

Recent large‐scale datasets, such as the ENIGMA Sleep consortium and WHO's global surveys on adolescent digital use, have provided important population‐level evidence on the associations between sleep patterns, digital engagement, and cognitive functioning (Boniel‐Nissim et al. 2024; Tahmasian et al. 2021). However, behavioral‐level investigations linking these factors to listening effort remain limited, particularly in smaller, controlled samples.

The relationships of sleep quality and digital addiction with listening effort are not clearly established in the literature. Cognitive difficulties compel individuals to expend more mental resources and may require greater effort to focus on the auditory message, accurately processing sounds (Francis and Love 2020). The division of attention across multiple tasks can strain cognitive capacity; however, participants may also have a tendency to reduce this load by automating simpler processes during dual or multitasking situations (Alhola and Polo‐Kantola 2007). Taken together, the literature suggests that both poor sleep quality and high levels of digital addiction may impair attention and working memory (key cognitive components underlying listening effort). Consequently, individuals experiencing lower sleep quality or higher digital addiction may need to allocate more cognitive resources during listening, resulting in greater listening effort.

The present study aimed to investigate the relationships between sleep quality, digital addiction, and listening effort in healthy young adults. Based on the literature, it was hypothesized that (1) sleep quality would be associated with listening effort, and (2) digital addiction levels would be associated with listening effort.

## 2 Materials and Methods

This study was approved by the Ethics Committee of Ankara Yildirim Beyazit University (26.09.2024, 07/866) and was conducted in accordance with the Declaration of Helsinki. Each participant signed an informed consent form indicating that participation was voluntary and that they could withdraw from the study at any time.

### 2.1 Participants

The study included voluntary individuals aged between 18 and 25 years who had normal hearing and general health and whose native language was Turkish. Individuals were excluded if they were outside the 18–25 age range, failed the hearing screening, were not native Turkish speakers, or reported any known general health issues that could interfere with the study. The hearing of the voluntary participants was screened via a screening audiometer, and informed consent was obtained from individuals who met the study inclusion criteria. A total of 60 participants were included in the study. Data were collected at the Audiology Laboratory of Ankara Yildirim Beyazit University.

### 2.2 Assessment

Listening effort was assessed via a dual‐task paradigm. Within this paradigm, repeating the auditorily presented sentence was designated the primary task, whereas a reaction time task served as the secondary task. During the test, the participants were instructed to prioritize the primary task. Sleep quality was measured via the Pittsburgh Sleep Quality Index (PSQI), and digital addiction was assessed via the Digital Addiction Scale (DAS). The participants practiced all the tasks before data collection began. To minimize potential learning and fatigue effects, randomization and controlled break periods were implemented during the listening effort dual‐task paradigm. The participants were given a 3‐min rest between each test block.

#### 2.2.1 Assessment of Listening Effort

**Sentence repetition (primary task)**: The Turkish Matrix Test (TMT) is a speech‐in‐noise test developed in 2015 (Zokoll et al. 2015). The sentences of the TMT were recorded by a professional female voice actor in a professional recording studio. Each sentence in the TMT is composed of 5 words with the following structure: subject‐number‐adjective‐object‐verb. The sentences are constructed by randomly selecting words from a 50‐word matrix. Owing to the large number of possible sentence combinations (100,000 in total) and the semantic incongruity of the sentences, sentence predictability is low. The audio stimuli were normalized for loudness and saved in WAV format. During the test, the prerecorded sentences were presented binaurally via headphones, and the participants were asked to repeat the sentences, with the total number of correctly repeated words being recorded by the researcher. Each list included 26 sentences. In the practice session before the test, 10 sentences were presented. A 5‐second pause was provided between sentences to allow participants to repeat them. The stimuli were presented at 70 dB via Sennheiser 400 S headphones.

**Reaction time task (secondary task)**: A modified version of the Stop‐Signal Task was used. Arrows pointing either left or right appeared randomly within a white circle. The participants were instructed to press the “N” key for right‐pointing arrows and the “B” key for left‐pointing arrows. Each arrow remained on the screen for a maximum of 500 ms. At the end of the task, the mean reaction time and error rate were calculated. Trials in which no key was pressed within 500 ms, reaction times below 150 ms, or incorrect key presses were classified as errors and excluded from the reaction time analysis. The test consisted of 96 trials. A practice session including 20 trials was conducted before the actual test. The task was implemented via the PsyToolKit platform (Stoet 2010, 2017).

Listening effort was evaluated by calculating the dual‐task effect (DTE). The dual‐task effect was determined via RT‐DTE and ER‐DTE data. The DTE was calculated via the following formula:

$$\begin{array}{ccc} & & \%RT-DTE\\ & & =\frac{\left(Dual\;task\;reaction\;time\;-\;Single\;task\;reaction\;time\right)}{Single\;task\;reaction\;time}\;\times \;100, \end{array}$$

$$\begin{array}{ccc} & & \%ER-DTE\\ & & =\frac{\left(Dual\;task\;error\;rate\;-Single\;task\;error\;rate\right)}{Single\;task\;error\;rate}\;\times \;100. \end{array}$$

#### 2.2.2 The Pittsburgh Sleep Quality Index

The Pittsburgh Sleep Quality Index (PSQI) is a 19‐item self‐report scale designed to assess sleep quality over a one‐month period in adults. It was developed by Buysse et al. (1989). A Turkish validity and reliability study was conducted by Ağargün et al. (1996). Each item is scored on a 0–3 scale, where 0 indicates “very good” and 3 indicates “very poor” sleep quality. The total PSQI score ranges from 0 to 21, with higher scores indicating poorer sleep quality. Permission for the use of the scale was obtained.

The participants were divided into two groups based on the mean PSQI score of the entire sample. The methodology was based on the procedure described by Sendesen et al. (2024). This approach allows for balanced group sizes and meaningful comparisons within the context of the current sample, accounting for population‐specific variations in sleep quality. Participants with scores above the mean were categorized into the high PSQI (H‐PSQI) group, whereas those with scores below the mean were categorized into the low PSQI (L‐PSQI) group.

#### 2.2.3 Digital Addiction Scale

To comprehensively assess the use of all digital devices (smartphones, tablets, computers, etc.), the Digital Addiction Scale (DAS) was used in this study. DAS was developed by Kesici and Tunç (2018). The scale consists of 19 items. The response options include “Totally Agree,” “Agree,” “Neither Agree nor Disagree,” “Disagree,” and “Totally Disagree,” which are scored as 5, 4, 3, 2, and 1, respectively. The total score is divided by the number of items to determine the participant's level of digital addiction. The possible score ranges from 1.00 to 5.00, with higher scores indicating higher levels of digital addiction. Permission for the use of the scale was obtained.

### 2.3 Statistical Analysis

Statistical analyses were conducted via SPSS version 21.0 (IBM Corporation, Armonk, NY). Numerical variables are expressed as the means (standard deviations) and medians (quartile 1, quartile 3). Categorical variables are reported as frequencies and percentages. The normality of the data distribution was assessed via the Shapiro‒Wilk test. Parametric tests (independent samples *t*‐test, paired samples *t*‐test, Pearson's correlation) were applied for normally distributed variables, whereas non‐parametric tests (Mann–Whitney *U* test, Wilcoxon test, Spearman's rho correlation) were used when normality assumptions were not met. Correlation analyses between sleep quality and listening effort, digital addiction and listening effort, and digital addiction and sleep quality were conducted via Pearson's and Spearman's rho tests. Differences between dependent groups were examined via the Wilcoxon test and the *t*‐test. A *p* value of less than 0.05 was considered statistically significant. Bayesian analyses were conducted using JASP (version 0.95.4).

An a priori power analysis was conducted using G*Power (version 3.1.9.7). Based on a desired power of 95% (1 – *β*), a significance level of 0.05 (*α*), and an expected effect size of *r* = 0.455 derived from our pilot study, a required sample of 52 participants was identified. Although the required minimum sample was determined to be 52, 60 participants were ultimately reached in order to increase the study's power and compensate for potential data loss (e.g., exclusion criteria, missing data).

## 3 Results

### 3.1 Demographic Characteristics

A total of 60 individuals participated in the study. Among the participants, 30 were female and 30 were male. The demographic characteristics of the participants are presented in Table 1.

**TABLE 1:** Demographic information.

	Female *n* = 30		Male *n* = 30	
**Age**	20.77 (±2.77)		19.10 (±1.86)	
**Education**	High school graduate Bachelor's degree Graduate degree **Total**	8 17 5 30	High school graduate Bachelor's degree Graduate degree **Total**	22 8 0 30

*n*: number of participants.

### 3.2 Comparisons of Listening Effort in Single and Dual Tasks

To evaluate listening effort, reaction time (RT), error rate (ER), and the number of correctly repeated words (CRW) were calculated. A statistically significant difference was found between the mean RT values of the single‐task and dual‐task conditions (*t* = 3.134, *p* = 0.003). RT significantly increased during the dual‐task condition. Similarly, there was a statistically significant difference between the error rates in the single‐task and dual‐task conditions (*Z* = 4.375, *p* < 0.001), with ER being significantly greater during the dual‐task condition. No statistically significant difference was found between the number of correctly repeated words in the single‐task and dual‐task conditions (*Z* = 1.849, *p* = 0.065). Comparative statistics between task conditions are summarized in Table 2.

**TABLE 2:** Listening effort measures.

	Single‐Task	Dual‐Task	*p*
RT	366.14 (±28.83)	373.80 (±22.11)	0.003^*^
ER	5.73 (3.13, 11.20)	11.46 (7.29, 19.53)	<0.001^**^
CRW	130 (129, 130)	130 (128, 130)	0.065^**^

^*^Paired sample *t*‐test. The results are expressed as mean (standard deviation).

^**^Wilcoxon signed rank test. The results are expressed as median (Q1, Q3).

RT: reaction time, ER: error rate, CRW: correctly repeated words, Q: quartile.

**FIGURE 1:**
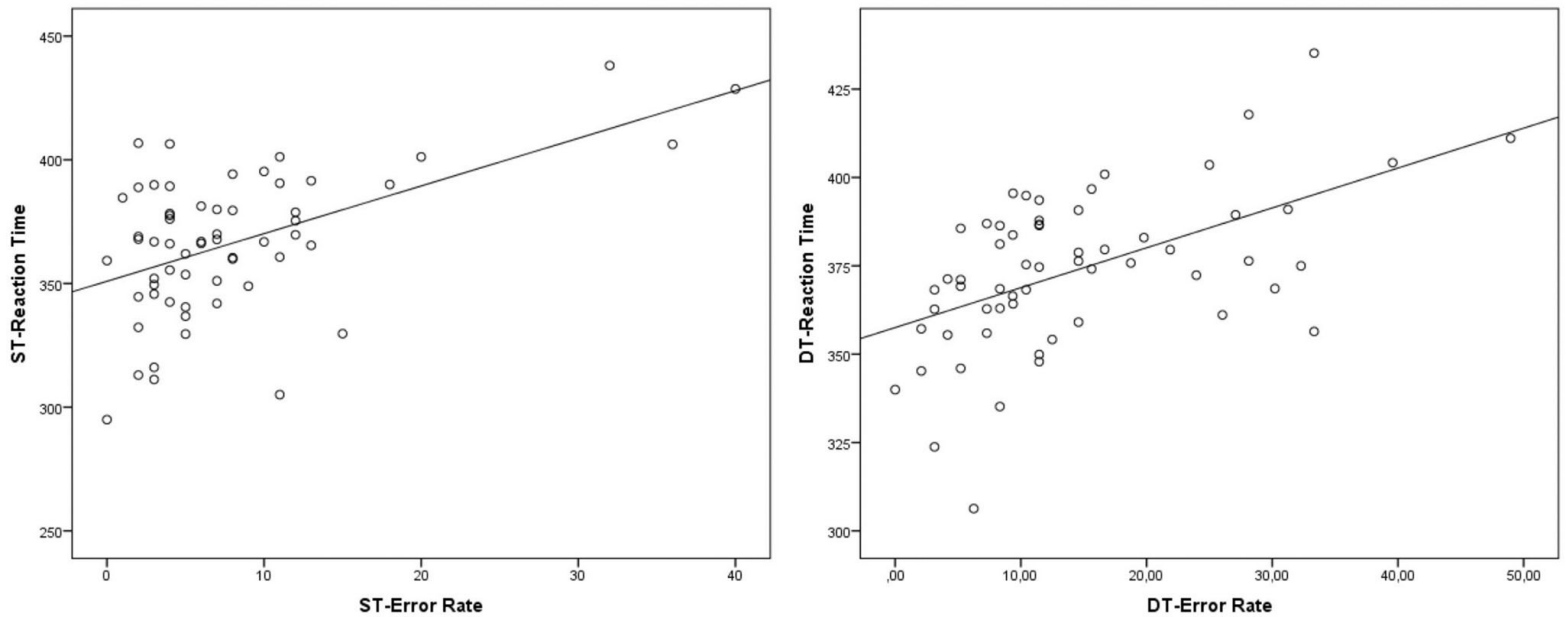
Scatter plot of the correlation between the ER and RT in the single‐task and dual‐task conditions. ST: single‐task, DT: dual‐task. The graphs were generated using SPSS version 21.0 (IBM Corporation, Armonk, NY) software.

There was a statistically significant weak positive correlation between the ER and RT in the single‐task condition (*r*_s_ = 0.360, *p* = 0.005). As the single‐task error rate increased, the reaction time also increased. A statistically significant moderate positive correlation was observed between the ER and RT in the dual‐task condition (*r*_s_ = 0.538, *p* < 0.001). As the dual‐task ER increased, the RT also increased (Figure 1).

### 3.3 Comparisons of the PSQI, DAS, and DTE Scores

The participants' mean score on the DAS was 3.14 (±0.70), and the mean PSQI score was 6.47 (±2.89). The mean RT‐DTE was 2.37% (±5.43), and the median ER‐DTE was 99.88% (0, 206.27).

There was no statistically significant correlation between the PSQI score and RT‐DTE score (*r*_s_ = 0.051, *p* = 0.699) or between the DAS score and RT‐DTE score (*r* = –0.091, *p* = 0.487). Similarly, no significant correlation was found between the PSQI score and the ER‐DTE score (*r*_s_ = 0.06, *p* = 0.541) or between the DAS score and the ER‐DTE score (*r*_s_ = 0.121, *p* = 0.367).

Bayesian correlation analyses revealed no meaningful associations between the variables. For RT‐DTE, neither the PSQI (Kendall's *τ* = –0.03, BF_10_ = 0.181; ≈5.6× evidence for no correlation) nor the DAS (Pearson's *r* = –0.091, BF_10_ = 0.204; ≈4.9× evidence for no correlation) showed evidence of a relationship. Similarly, for ER‐DTE, both the PSQI (Kendall's *τ* = 0.060, BF_10_ = 0.213; ≈4.7× evidence for no correlation) and the DAS (Kendall's *τ* = –0.080, BF_10_ = 0.252; ≈4.0× evidence for no correlation) provided greater support for the absence rather than the presence of an association.

After controlling for age and gender, the partial correlation analysis revealed no significant associations between DAS and ER‐DTE (*r* = –0.156, *p* = 0.250), DAS and RT‐DTE (*r* = –0.023, *p* = 0.868), PSQI and ER‐DTE (*r* = –0.040, *p* = 0.768), or PSQI and RT‐DTE (*r* = –0.085, *p* = 0.533).

The mean PSQI scores for the H‐PSQI and L‐PSQI groups were 9.00 (±2.00) and 4.25 (±1.24), respectively. There was no statistically significant difference in RT‐DTE scores between the H‐PSQI and L‐PSQI groups (*t* = 1.654, *p* = 0.104). Two participants with an error rate of zero in the single‐task condition were excluded from the ER‐DTE analysis. No statistically significant difference was detected between the H‐PSQI and L‐PSQI groups in terms of ER‐DTE scores (*Z* = 0.383, *p* = 0.702) (Table 3).

**TABLE 3:** Comparisons of listening effort by sleep quality group.

	PSQI Groups		
	L‐PSQI	H‐PSQI	*p*
RT‐DTE	3.43 (±5.90) (*n* = 32)	1.15 (±4.63) (*n* = 28)	0.104^*^
ER‐DTE	91.68 (0, 193) (*n* = 32)	99.94 (–4.16, 274.72) (*n* = 26)	0.702^**^

^*^Independent samples *t*‐test. The RT‐DTE results are expressed as mean (standard deviation).

^**^Mann‒Whitney *U* test. The ER‐DTE results are expressed as median (Q1, Q3).

PSQI: Pittsburgh Sleep Quality Index, L‐PSQI: Low PSQI, H‐PSQI: High PSQI, RT‐DTE: reaction time dual task effect, ER‐DTE: error rate dual task effect, Q: quartile, *n*: number of participants.

A Bayesian independent samples *t*‐test showed no clear difference in RT‐DTE scores between the L‐PSQI and H‐PSQI groups (BF_10_ = 0.820; BF_01_ ≈1.22, anecdotal evidence for the null). A Bayesian Mann–Whitney *U* test indicated no group difference in ER‐DTE (BF_10_ = 0.276; ≈3.6× evidence for no difference).

## 4 Discussion

This study examined the relationships of sleep quality and digital addiction with listening effort, which was successfully measured using a dual‐task paradigm demonstrated by a significant difference in reaction times and error rates between the single‐task and dual‐task conditions. The primary analyses indicated no significant relationship between listening effort and either sleep quality or digital addiction. Specifically, no significant difference in listening effort was observed among the different sleep quality groups.

Our findings, indicating no significant relationship between listening effort and either sleep quality or digital addiction, can be interpreted within competing theoretical frameworks. According to the limited capacity model (Pichora‐Fuller et al. 2016), cognitive resources are finite; however, in the present study, the listening task may not have been demanding enough to exceed participants’ resource limits. Conversely, as suggested by Eysenck (1982) in his discussion of arousal and performance, elevated arousal or alertness may temporarily compensate for resource limitations. In this context, factors such as digital engagement or mild sleep restriction can influence arousal, thereby maintaining performance levels despite potential cognitive strain. As a result, individuals may maintain their performance despite an increased mental load. Thus, null results do not necessarily indicate the absence of an effect but may reflect dynamic resource allocation processes under varying arousal states. Thus, the null results observed in this study may reflect both the possibility that no meaningful associations exist under the current task demands and the presence of compensatory arousal‐related mechanisms that stabilize performance. Additionally, the Bayesian analyses provided moderate evidence in favor of the null hypothesis, strengthening the interpretation that any potential effects, if present, were likely small or undetectable under the present experimental conditions.

Sleep is linked to various cognitive functions, such as attention, working memory, executive functions, and processing speed (Alhola and Polo‐Kantola 2007). Although the effect of sleep quality on cognitive processes is known, no correlation between sleep and listening effort was found in this study. This lack of correlation provides an important context for the sleep‐cognition mechanism. The literature indicates a negative relationship between sleep quality and mental fatigue (Pastier et al. 2022). In our study design, we aimed to control the effect of acute, task‐induced fatigue by incorporating standardized rest periods between tasks. Thus, our inability to find a correlation suggests that chronic deficits resulting from subjective poor sleep quality do not measurably increase listening effort when acute fatigue is controlled. This result suggests that listening effort may be resilient to the effects of moderate sleep quality, provided that task‐specific fatigue is minimized.

Despite research indicating that auditory attention is impacted by sleep deprivation, it is noted that sleep deprivation does not uniformly affect individuals and that individual differences are significant factors (Linde et al. 1999). Similarly, sleep quality is also influenced by individual differences (Van Dongen et al. 2005). This suggests that the relationship between sleep quality and listening effort might be indirect or variable depending on individual differences. Few studies in the literature have investigated the relationship between sleep quality and auditory processing. However, studies have demonstrated the effects of sleep deprivation on attention and auditory processing. PSQI consists of questions assessing sleep duration, problems experienced during sleep, and the extent to which sleep affects daily life. Although sleep deprivation is a parameter affecting sleep quality, it is not the sole determinant of overall sleep quality.

Sleep deprivation, as one of the factors affecting sleep quality, negatively affects selective attention, leads to an increase in reaction times, and causes behavioral instability (Kirszenblat and van Swinderen 2015). Sleep affects selective attention; however, auditory processing capabilities are substantially maintained (Kirszenblat and van Swinderen 2015). However, sleep‐deprived individuals showed increased response times to auditory stimuli. This observation could be due to reduced attention and alertness rather than a direct impairment in auditory processing (Horne et al. 1983). Auditory‐temporal resolution, which is dependent on the function of the prefrontal cortex, a region particularly sensitive to sleep deprivation, is negatively affected by sleep deprivation (Babkoff et al. 2005). Measuring the effect of 35 h of sleep deprivation on cognitive performance and brain activity, Drummond et al. did not observe impairment in participants' attention processes (Drummond et al. 2001). In a comprehensive experimental study conducted on healthy young adults, no relationships were reported between subjective sleep quality and working memory or executive functions (Zavecz et al. 2020).

The correlation analysis revealed no significant relationship between the DAS scores and listening effort. This finding suggests that addiction levels are not linearly related to the cognitive effort expended during listening. In this context, changes in the amplitude and latency of the P300, which is considered a physiological and objective indicator of attention and memory processes that contribute to listening effort, become significant (Kestens et al. 2023). Indeed, some studies have reported a significant correlation between the P300 amplitude and the severity of internet gaming disorder (IGD) symptoms (Park et al. 2016). These findings suggest that this disorder can cause impairments in auditory information processing and cognitive functions. Although these findings suggest that digital addiction could affect cognitive functions, there is no consistent data concerning its impact on listening effort.

Studies investigating the impact of digital media use on central auditory processing have reported similar findings. In a study using the Amsterdam Inventory for Auditory Disability and Handicap, Söylemez et al. (2025) reported that while all participants had normal hearing, the group with high smartphone use demonstrated poorer central auditory processing skills. Another study suggested that long‐term mobile phone use could be a risk factor for tinnitus (Hutter et al. 2010). Sendesen et al. (2023) noted that listening effort could be elevated in individuals experiencing tinnitus symptoms. However, these studies indicate the presence of other factors influencing listening effort, thereby suggesting that the effect of digital device use might be indirect.

## 5 Conclusion

To the best of our knowledge, this is the first study to examine listening effort from the perspectives of sleep quality and digital addiction. In conclusion, this study demonstrates that listening effort in healthy young adults is not significantly influenced by sleep quality or digital addiction under controlled task conditions. The absence of a relationship between sleep quality and listening effort, despite the link between sleep quality and fatigue, supports the notion that the measured outcomes genuinely reflect listening effort, particularly since fatigue was minimized during measurements. Although the impact of sleep quality on cognitive processes is well established, the findings suggest that listening effort may be resilient to sleep quality and digital addiction. The results of this study indicate that digital addiction and sleep quality do not have a statistically significant effect on listening effort, suggesting that interventions aimed at reducing listening effort should primarily focus on other cognitive and auditory factors. However, the lack of significant results may also indicate that performance was maintained due to factors such as task difficulty, individual differences, or levels of arousal. Despite its contribution to the literature, our study highlights the value of conducting further research on this topic.

Limitations of the present study include the fact that listening effort was not measured in noise, and that only young adults were included in the study sample. Furthermore, the use of subjective scales (DAS and PSQI) introduces limitations. In this type of self‐report scale, factors such as individual differences, response bias, level of insight, and honesty can influence the obtained results. Another limitation concerns the potential floor and ceiling effects observed in RT‐DTE and ER‐DTE measures. This variability may reflect individual performance differences and the bounded nature of the measures. The wide variability in these scores may have reduced the sensitivity of the dual‐task paradigm, potentially obscuring subtle associations between listening effort, sleep quality, and digital addiction. Although no linear relationship was observed between addiction levels and listening effort, the possibility of non‐linear associations cannot be ruled out and could be explored in future studies using appropriate non‐linear methods. Future studies incorporating broader age ranges, utilizing objective measurement methods, and accounting for different listening conditions will contribute to a more comprehensive evaluation of these findings.

## Author Contributions

**Banu Baş**: conceptualization, investigation, methodology, writing – review and editing, resources, project administration, supervision. **Elif Zehra Biber**: conceptualization, investigation, methodology, data curation, formal analysis, visualization, writing – original draft, resources. **Gülse Akdemir**: investigation, resources, data curation, formal analysis, visualization, writing – original draft, methodology.

## Funding

The authors have nothing to report.

## Ethics Statement

Approval was granted by the Ethics Committee of Ankara Yildirim Beyazit University (26.09.2024, 07/866). The study was conducted in accordance with the Declaration of Helsinki. Informed consent was obtained from all individual participants included in the study.

## Conflicts of Interest

The authors declare no conflicts of interest.

## Data Availability

The data that support the findings of this study are available on request from the corresponding author. The data are not publicly available due to privacy or ethical restrictions.
